# Epidemiology, Clinical Features and Prognostic Factors of Pediatric SARS-CoV-2 Infection: Results From an Italian Multicenter Study

**DOI:** 10.3389/fped.2021.649358

**Published:** 2021-03-16

**Authors:** Silvia Garazzino, Andrea Lo Vecchio, Luca Pierantoni, Francesca Ippolita Calò Carducci, Federico Marchetti, Antonella Meini, Elio Castagnola, Gianluca Vergine, Daniele Donà, Samantha Bosis, Icilio Dodi, Elisabetta Venturini, Enrico Felici, Roberta Giacchero, Marco Denina, Luca Pierri, Giangiacomo Nicolini, Carlotta Montagnani, Andrzej Krzysztofiak, Sonia Bianchini, Caterina Marabotto, Pier-Angelo Tovo, Giulia Pruccoli, Marcello Lanari, Alberto Villani, Guido Castelli Gattinara, The Italian SITIP-SIP Pediatric Infection Study Group, Valeria Caldarelli

**Affiliations:** ^1^Pediatric Infectious Diseases Unit, Regina Margherita Children's Hospital, University of Turin, Turin, Italy; ^2^Section of Pediatrics, Department of Translational Medical Science, University of Naples Federico II, Naples, Italy; ^3^Pediatric Emergency Unit, IRCCS Azienda Ospedaliero-Universitaria di Bologna, Bologna, Italy; ^4^Universitarian-Hospital Department, Ospedale Bambino Gesù IRCCS, Rome, Italy; ^5^Department of Pediatrics, Santa Maria delle Croci Hospital, Ravenna, Italy; ^6^Department of Experimental and Clinical Sciences, Pediatric Clinic, University of Brescia, Brescia, Italy; ^7^Infectious Diseases Unit, IRCCS Istituto Giannina Gaslini, Genoa, Italy; ^8^UOC Pediatria, Ospedale degli Infermi di Rimini, Rimini, Italy; ^9^Division of Pediatric Infectious Diseases, Department of Women's and Children's Health, University Hospital of Padua, Padua, Italy; ^10^Fondazione IRCCS Cà Granda Ospedale Maggiore Policlinico, Milan, Italy; ^11^Emergency and General Pediatric Unit, Pietro Barilla Children's Hospital, Parma, Italy; ^12^Infection Disease Unit, Meyer Children's University Hospital, Florence, Italy; ^13^Pediatric and Pediatric Emergency Unit, The Children Hospital, AO SS Antonio e Biagio e C. Arrigo, Alessandria, Italy; ^14^UOC Pediatria ASST di Lodi, Lodi, Italy; ^15^UOC Pediatria, San Martino Hospital, Belluno, Italy; ^16^Department of Pediatrics, ASST Santi Paolo e Carlo Hospital, Milan, Italy

**Keywords:** SARS-CoV-2 infection, children, multisystem inflammatory syndrome, MIS-C, COVID-19

## Abstract

**Background:** Many aspects of SARS-CoV-2 infection in children and adolescents remain unclear and optimal treatment is debated. The objective of our study was to investigate epidemiological, clinical and therapeutic characteristics of pediatric SARS-CoV-2 infection, focusing on risk factors for complicated and critical disease.

**Methods:** The present multicenter Italian study was promoted by the Italian Society of Pediatric Infectious Diseases, involving both pediatric hospitals and general pediatricians/family doctors. All subjects under 18 years of age with documented SARS-CoV-2 infection and referred to the coordinating center were enrolled from March 2020.

**Results:** As of 15 September 2020, 759 children were enrolled (median age 7.2 years, IQR 1.4; 12.4). Among the 688 symptomatic children, fever was the most common symptom (81.9%). Barely 47% of children were hospitalized for COVID-19. Age was inversely related to hospital admission (*p* < 0.01) and linearly to length of stay (*p* = 0.014). One hundred forty-nine children (19.6%) developed complications. Comorbidities were risk factors for complications (*p* < 0.001). Viral coinfections, underlying clinical conditions, age 5–9 years and lymphopenia were statistically related to ICU admission (*p* < 0.05).

**Conclusions:** Complications of COVID-19 in children are related to comorbidities and increase with age. Viral co-infections are additional risk factors for disease progression and multisystem inflammatory syndrome temporarily related to COVID-19 (MIS-C) for ICU admission.

## Introduction

Since December 2019 Coronavirus Disease (COVID-19) caused by severe acute respiratory syndrome coronavirus 2 (SARS-CoV-2) has rapidly spread, becoming the first pandemic of the 21st century for number of deaths (1). From the available data, children appear to be less affected than adults, with a significantly lower mortality rate, although severe complications may also occur (2–6). However, many aspects of SARS-CoV-2 infection in children and adolescents remain unclear, optimal treatment is debated and the role of young children as drivers of viral transmission is under discussion.

We hereby report the results of a national multicentre study aimed at investigating epidemiological, clinical and therapeutic aspects of SARS-CoV-2 infection in infants, children and adolescents, hereafter referred to as pediatric population or children.

## Materials and Methods

The present study was promoted by the Italian Society of Pediatric Infectious Diseases, with the endorsement of the Italian Society of Pediatrics and involved 11 of 13 exclusively pediatric hospitals, 51 pediatric units across Italy as well as general pediatricians. All subjects with < 18 years of age with documented SARS-CoV-2 infection were recruited. Diagnosis of infection was established in presence of at least one respiratory specimen positive for SARS-CoV-2 nucleic acid using a validated real-time reverse-transcriptase polymerase-chain-reaction (RT-PCR) assay. All molecular tests fulfilled performance criteria established by the European Commission (7) and tested envelope protein gene (E), nucleocapsid protein gene (N) and RNA-dependent RNA polymerase gene (RdRp). Only highly suggestive symptomatic children with COVID-positive households were diagnosed through detection of IgM and IgG antibodies against SARS-CoV-2 (DiaSorin Inc.) if molecular testing was not available (as occasionally occurred in March-April due to shortage of reagents). Children were generally retested after 14 days from the first positive molecular test, and then weekly until a negative result.

Medical records of all enrolled patients were revised as of 15 September 2020. Data were de-identified, recorded through a targeted registration form and transferred to a specifically designed database. Data collection was allowed by written consent of at least one parent for active participation to the study.

The study received ethical approval on 24 March 2020 (protocol number 0031296).

An adequate follow-up to outline the outcome of the infection was required, in most instances at least 2 weeks.

Children were categorized into 3 groups according to the healthcare setting: children hospitalized for COVID-19-related reasons; children with SARS-CoV-2 infection but hospitalized for reasons other than COVID-19; outpatients, including children entirely followed by their general pediatrician or children visited and tested in the emergency department but not hospitalized.

The COVID-19-related complications were defined as follows: (a) clinical and/or radiological diagnosis of pneumonia; (b) severe acute respiratory illness (SpO2 < 92% associated with tachypnea and other signs of respiratory failure); (c) acute respiratory distress syndrome; (d) neurological disturbances; (e) severe dehydration requiring intravenous rehydration; (f) severe bacterial supra-infection; (g) specific involvement of a single organ/apparatus requiring hospitalization (i.e., myocarditis, pericarditis, pancreatitis, etc.); (h) multisystem inflammatory syndrome temporarily related to COVID-19 (MIS-C) according to CDC criteria (8).

Statistical analysis was performed using IBM SPSS Statistics 25.0 (IBM Corp. Armonk, NY). Statistical significance was set at *p* < 0.05. All *p*-values were 2-tailed. In the descriptive analysis categorical variables were expressed as percentage and continuous variables as mean, median and interquartile range (IQR). To compare continuous variables of the study groups, Student *t*-test was used. To evaluate discrete variables, Pearson χ^2^ and correlation Fisher exact tests were performed as appropriate. One-way analysis of variance (ANOVA) was used for linear regression analysis.

## Results

As of 15 September 2020, 759 children were enrolled (426, 56.1%, males—Table 1). The mean age was 7.3 years (median 7.2 years, IQR: 1.4–12.4 years). Of them, 160 (21.1%) were infants, including 40 neonates (3 preterm).

**Table 1:** Characteristics of enrolled children according to health-care setting.

		**Total (*n* = 759)**	**Outpatients (*n* = 371, 48.9%)**	**Inpatients (*****n*** **= 388, 51.1%)**	
				**Admitted for COVID-19 *(n* = 361)**	**Admitted for other reasons (*n* = 27)**
**Age (years)**					
Mean, median (IQR)		7.3, 7.2 (1.4–12.4)	8.6, 9.2 (3.7–13.2)	5.8, 3.8 (0.5–10.8)	8.7, 9.9 (3.8–12.6)
**Age groups**					
<1 y		160 (21.1%)	38 (10.2%)	117 (32.4%)	6 (22.2%)
1–4 y		160 (21.1%)	78 (21%)	80 (22.2%)	;2 (7.4%)
5–9 y		155 (20.4%)	87 (23.4%)	63 (17.4%)	6 (22.2%)
10–17 y		284 (37.4%)	168 (45.3%)	101 (28.0%)	13 (48.2%)
**Gender**					
Males		426 (56.1%)	205 (55.3%)	207 (57.3%)	13 (48.2%)
Females		333 (43.9%)	166 (44.7%)	154 (42.7%)	14 (51.8%)
**Underlying chronic diseases**					
Total		136 (17.9%)	50 (36.8%)	80 (58.8%)	6 (4.4%)
Congenital malformations		20 (14.7%)	3 (6%)	17 (21.3%)	/
Asthma		15 (11.0%)	8 (16%)	7 (8.8%)	/
Epilepsy		15 (11.0%)	8 (16%)	7 (8.8%)	/
Complex genetic syndromes		13 (9.6%)	4 (8%)	9 (11.2%)	/
Endocrine disorders		11 (8.1%)	5 (10%)	3 (3.7%)	3 (50%)
Cancers		9 (6.6%)	2 (4%)	7 (8.8%)	/
Hematologic diseases		9 (6.6%)	4 (8%)	5 (6.2%)	/
Rheumatologic diseases		8 (5.9%)	4 (8%)	4 (5%)	/
Autism or neurological development impairment		8 (5.9%)	4 (8%)	4 (5%)	/
Gastrointestinal diseases		6 (4.4%)	3 (6%)	3 (3.7%)	/
Genitourinary diseases		6 (4.4%)	3 (6%)	3 (3.7%)	/
Cystic fibrosis and other chronic lung diseases		5 (3.7%)	1 (2%)	4 (5%)	/
Metabolic disorders		4 (2.9%)	/	3 (3.7%)	1 (16.7%)
Hydrocephalus		3 (2.2%)	/	1 (1.3%)	2 (33.3%)
Severe obesity		2 (1.5%)	/	2 (2.5%)	/
Otolaryngologic diseases		2 (1.5%)	1 (2%)	1 (1.3%)	/
**Signs and symptoms (*****n*** **=** **668)**					
Fever		547 (81.9%)	247	292	8
Cough		254 (38.0%)	124	126	4
Rhinitis		139 (20.8%)	62	77	/
Diarrhea		107 (16.0%)	41	64	2
Pharyngodynia/pharyngitis		86 (12.9%)	39	45	2
Vomiting		67 (10.0%)	16	48	3
Headache		67 (10.0%)	59	8	/
Dyspnea		62 (9.3%)	2	60	/
Hyporexia		60 (9.0%)	22	38	/
Conjunctivitis		56 (8.4%)	30	26	/
Fatigue		55 (8.2%)	45	10	/
Abdominal pain		52 (7.8%)	18	32	2
Skin rash		38 (5.7%)	13	25	2
Smell and taste alterations		27 (4.0%)	19	8	1
Arthromyalgy		23 (3.4%)	17	6	/
Chest pain		16 (2.4%)	6	10	/
Febrile seizures		11 (1.6%)	/	11	/
Non-febrile seizures in known epilepsy		3 (0.5%)	/	2	1

The majority of children (535/759, 70.5%) had at least one infected parent, with the mother being carrier in 60% of cases. Close contact with other infected households was reported in 76 (10.0%) subjects; 377 children (49.7%) had more than one infected family member.

Of the 569 children with known immunization status, only 26 (4.6%) had received seasonal 2019–2020 flu vaccine. One hundred and thirty-six children (17.9%) had underlying chronic diseases (Table 1); forty (5.3%) had a history of premature birth.

### Clinical Presentation

Ninety-one (12.0%) children were asymptomatic: they were screened either for a contact with a SARS-CoV-2 positive person or during hospitalization for other reasons. None developed symptoms at follow-up.

**Figure 1:**
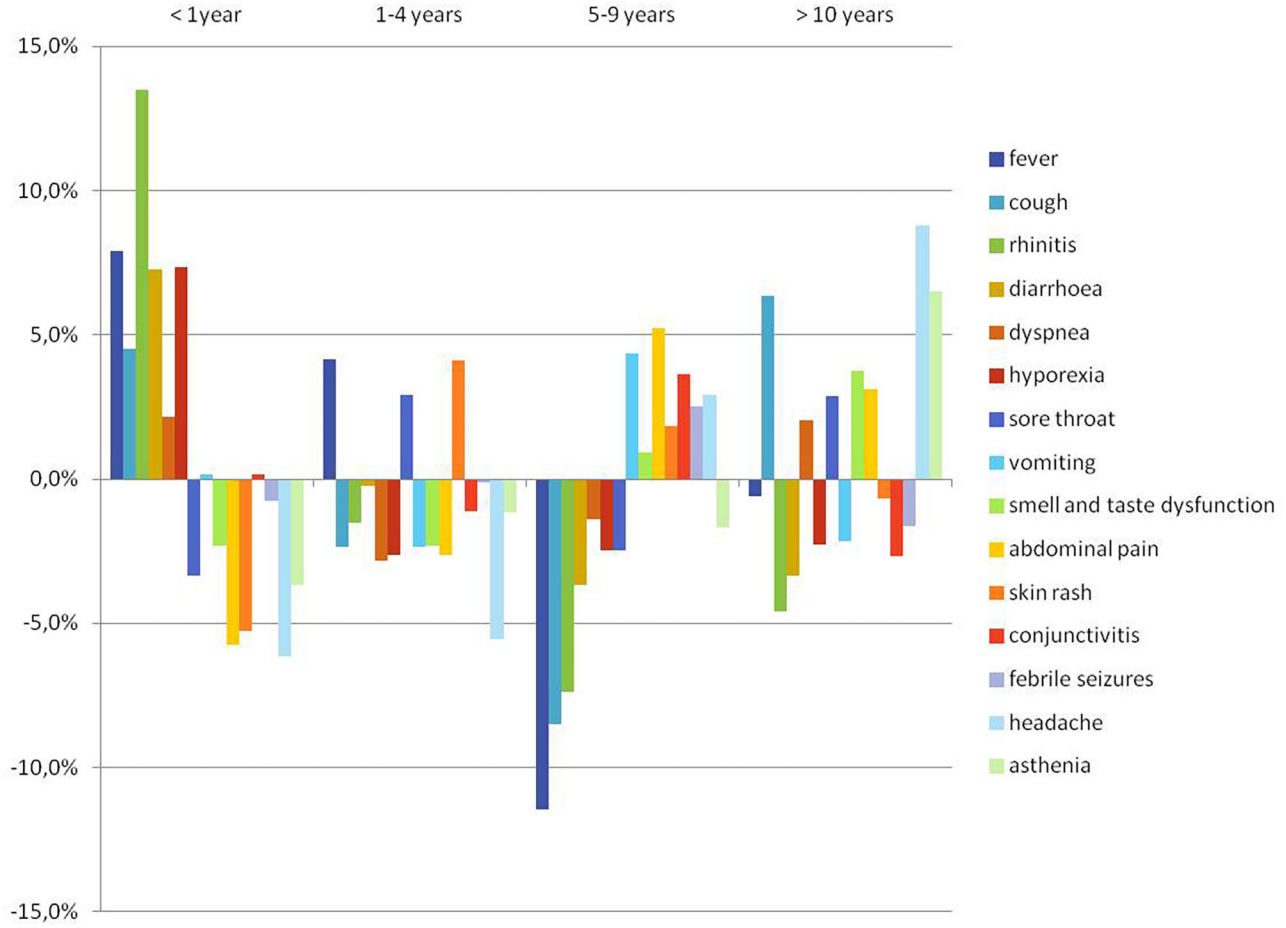
COVID-19-related clinical manifestations according to age.

Among the 668 (88.0%) symptomatic children, the pattern of presentation of COVID-19 differed by age (Figure 1). Fever (37.5–40 °C) was the most common symptom (81.9%), followed by cough and rhinitis (Table 1). Respiratory disturbances were reported in 347 patients (51.9%), with dyspnea in 62. Gastrointestinal symptoms were observed in 174 patients (26.0%).

Infants were significantly more likely to present with fever, rhinitis and/or diarrhea and/or hyporexia; on the contrary, headache, asthenia, cough, abdominal pain and smell and taste alterations showed an increasing frequency trend with age (Figure 1).

The median interval between symptom onset and first medical evaluation was 2 days (IQR 1;5).

### Co-infections

Concomitant infecting pathogens were not systematically searched for: 244 children (32.1%) were tested for concomitant infecting pathogens and a co-infection was found in 69 (28.3%); of these, 8 (11.6%) had multiple infections. Agents responsible for co-infection were viruses in 45 cases and bacteria in 32; in particular, respiratory viruses were Rhino/Enterovirus, Adenovirus, Bocavirus, Respiratory Syncytial Virus, Coronavirus other than SARS-CoV-2, Influenza A/B and Parainfluenza virus.

### Hospital Admission and COVID-19 Complications

Barely a half of children (388/759) were hospitalized (Table 1). The vast majority (361/388, 93.0%) was admitted for COVID-19-related reasons; of these, only 25 (6.9%) were referred to hospital by a pediatrician or family doctor. Children admitted for other reasons with an incidental diagnosis of SARS-CoV-2 infection were excluded from the analysis on risk factors for complicated disease.

Median duration of hospital stay of children admitted for COVID-19 was 6 days (IQR 4;11, mean 8.72 days). Hospital stay was longer in the first phase of the pandemic (March-April) than in the following months, though without statistical significance (mean 8.94 *vs*. 7.42 days, respectively, *p* = 0.14).

Age was inversely related to hospital admission (*p* < 0.01) and linearly related to length of stay (*p* = 0.014). Hospital admission and length of stay were not influenced by gender (*p* = 0.27 and *p* = 0.42, respectively). The hospitalization rate was significantly higher among ex-preterm (*p* = 0.014) and among children with co-morbidities or immunodeficiency (*p* < 0.01 and 0.009, respectively).

One hundred and forty-nine children (19.6% of the entire study population and 38.8%, 140/361, of those hospitalized for COVID-related causes) developed one or more complications. Mean age was significantly higher among children with complications than in uncomplicated infections (6.9 *vs*. 5.5 years, respectively; *p* = 0.023), while gender was non-contributory to complications development (Table 2). At multivariate analysis, fever or abdominal pain at onset and co-morbidities were risk factors for complications (Table 3).

**Table 2:** Summary of the main results at univariate analysis.

**Variable**	**Hospital admission for COVID-19 related reasons**			**Complicated SARS-CoV-2 infection**			**Ventilatory support**			**ICU admission**		
	**Yes**	**No**	***p***	**Yes**	**No**	***p***	**Yes**	**No**	***p***	**Yes**	**No**	***p***
Age (median)	5.8	8.6	**<0.001**	7.2	7.3	0.86	6.6	7.4	0.38	7.7	7.3	0.68
**Age group**												
<1year	117	38	**<0.001**	35	125	0.83	12	148	0.25	5	155	
1–4 years	80	78		32	128		8	152		2	158	
5–9 years	63	87		30	125		13	142		11	144	**0.039**
>10 years	101	168		52	232		12	272		9	275	
**Demographics**												
Male gender	207	205	0.57	83	343	0.9	26	400	0.81	16	410	0.73
Underlying clinical conditions	80	50	**<0.001**	47	89	**<0.001**	23	113	**<0.001**	14	122	**<0.001**
Immunosuppression	13	4	**0.021**	4	13	0.68	0	17	0.29	1	16	0.6
None/only one infected family member	217	153	**<0.001**	104	279	**<0.001**	39	344	**<0.001**	23	360	**<0.001**
**Clinical manifestations**												
Fever	292	247	**<0.001**	125	422	**<0.001**	40	507	**0.01**	25	522	**0.016**
Abdominal pain	32	18	**0.026**	24	28	**<0.001**	7	45	**0.017**	4	48	0.095
**Laboratory results**												
Leukocytosis	65	8	**0.024**	44	34	**<0.001**	18	60	**<0.001**	11	67	**0.001**
Lymphopenia	65	16	0.8	46	36	**<0.001**	20	62	**<0.001**	19	63	**<0.001**
Viral coinfection	44	1	**0.016**	21	24	0.87	13	32	**0.015**	8	37	**0.009**

*ICU, intensive care unit. Bold values are statistically significant*.

**Table 3:** Risk factors for hospital admission, complications, ventilatory support, and ICU admission at multivariate analysis.

**Variable**	**Hospital admission**			**Complications**			**Ventilatory support**			**ICU admission**		
	***p***	**OR**	**95% CI**	***p***	**OR**	**95% CI**	***p***	**OR**	**95% CI**	***p***	**OR**	**95% CI**
Gender	0.714	0.94	0.69–1.30	0.267	0.80	0.54–1.18	0.574	0.83	0.43–1.61	0.794	0.90	0.40–2.03
Age	**<0.001**	1.11	1.08–1.14	0.297	1.02	0.98–1.05	0.087	1.05	0.99–1.12	0.945	1.00	0.93–1.08
Chronic comorbidities	**0.002**	2.03	1.29–3.18	**<0.001**	3.01	1.88–4.81	**<0.001**	6.77	3.40–13.45	**<0.001**	4.91	2.11–11.42
More than one infected family member	**<0.001**	0.48	0.35–0.66	**<0.001**	0.38	0.26–0.58	**<0.001**	0.17	0.07–0.42	**0.004**	0.20	0.07–0.61
Fever	**<0.001**	2.36	1.64–3.38	**<0.001**	2.58	1.57–4.24	**0.012**	3.55	1.32–9.51	**0.026**	5.32	1.23–23.10
Abdominal pain	**0.001**	3.11	1.62–5.96	**<0.001**	5.37	2.82–10.19	**0.012**	3.65	1.33–9.99	**0.098**	2.75	0.83–9.12
Immunosuppression	0.109	2.76	0.80–9.57	0.466	0.63	0.19–2.16	0.998	0.00	0.00	0.715	0.67	0.08–5.80

*OR, odds ratio; CI, confidence interval; ICU, intensive care unit. Bold values are statistically significant*.

Respiratory complications were reported in 123 children (16.2%) and included pneumonia (*n* = 109), severe acute respiratory illness (*n* = 50) and acute respiratory distress syndrome (*n* = 5). Three-hundred children (39.5%) underwent chest X-ray, with a normal finding in 148, pulmonary consolidations and/or interstitial involvement in the remaining and pleural effusions in 17. A chest computed tomography (CT) scan was performed in 28 children (3.7%).

Neurological complications were recorded in 5 patients (4 worsening of known epilepsy and 1 development of autoimmune encephalitis), while none had SARS-CoV-2-driven encephalitis.

Gastroenterological complications occurred in 13 children: mesenteritis or appendicitis in 9 (2 underwent appendectomy), pancreatitis in 1, and severe dehydration consequent to vomiting or diarrhea in 3.

Isolated cardiac involvement, including myocarditis and/or pericarditis, was documented in 7 (0.9%) children.

Six (0.8%) had a severe bacterial complication: five sepsis and one a retropharyngeal abscess.

MIS-C occurred as a complication of SARS-CoV-2 infection in 30 children (3.9%) with a median age of 6.6 years (IQR 3.4; 9.6). The development of MIS-C was unrelated to gender, although children with MIS-C were mainly males (21/30, 70.0%, *p* = 0.60). Eight children with MIS-C had co-morbidities, including one with immune deficiency (Wiskott–Aldrich syndrome). Fever, gastrointestinal disturbances, rash and conjunctivitis were the most common findings at MIS-C onset, followed by sore throat, cough and dyspnea. Overall, 17/30 patients (56.6%) had signs of cardiovascular involvement, with arrhythmia, left ventricle dysfunction, coronary aneurysms and pericardial effusions. Eleven patients (36.7%) developed acute respiratory failure. Gastrointestinal manifestations, including severe abdominal pain and mesenteritis, occurred in 17 patients (56.7%), while 5 (16.7%) had transient neurological deterioration following a decline in general condition. Eleven (36.6%) required respiratory support and two needed vasoactive medications.

### ICU Admission

Thirty children (4.0%) required intensive care support for COVID-19. ICU admission in the MIS-C group was 6 times higher than in other children: 20.0% (6/30) vs. 3.3% (24/729, *p* < 0.01), respectively. Overall median ICU stay was 6 days (IQR 6;11, range 1–47) vs. 7 days in the MIS-C group.

Viral co-infections, underlying clinical conditions and age between 5 and 9 years were statistically related to ICU admission (Table 2). No association was found with gender. At multivariate analysis, the presence of co-morbidities was confirmed as a risk factor for the need of intensive care support (Table 3).

Three patients admitted to ICU required intubation and mechanical ventilation. Two children underwent extracorporeal membrane oxygenation: the first for severe respiratory distress in pneumonia, known epilepsy and neurodevelopmental delay, the second for severe ARDS and concomitant sickle-cell disease. Five children were treated with C-PAP and 9 with high flow nasal cannulae oxygen therapy.

The need for ventilator support was statistically related to the presence of underlying chronic diseases (Tables 2, 3).

Having more than one infected family member had a protective role in terms of complications, hospital and ICU admission and need for ventilatory support.

### Laboratory Results

In children who underwent blood investigations (454/759, 59.8%), an increase in C-reactive protein (CRP) was the most common finding (39.5%), while other alterations were less frequently observed. Table 4 shows lab tests performed in patients admitted for COVID-19. D-dimer and troponin T were performed only in more severe cases. Children who needed ventilatory support and those with MIS-C were more likely to have a low lymphocytes count and/or a high CRP level as compared to children with mild-moderate disease or pneumonia (*p* < 0.005). Children with MIS-C also had significantly higher ESR (*p* < 0.001), ferritin (*p* < 0.001) and ALT (*p* = 0.001).

**Table 4:** Laboratory results of patients hospitalized for COVID-19: comparison of mild-moderate vs. severe forms requiring respiratory support.

**Laboratory test**	**Total** **n. abnormal/n. tested**	**Mild-moderate disease** **n. abnormal/n. tested**	**Ventilatory support** **n. abnormal/n. tested**	***p***
CRP (> 10 mg/L)	196/318	99/283	27/35	**<0.001**
Ferritin (> 150 ng/mL)	28/178	19/159	9/19	**0.005**
ESR (> 20 mm/h)	25/194	18/179	7/15	**0.006**
Leukocytes (higher than ULN according to age limits)	57/319	43/284	14/35	**0.009**
Lymphocytes (lower than ULN according to age limits)	49/319	36/284	13/35	**0.005**
ALT (> 2 × ULN)	39/307	32/272	7/35	0.103

*N, number; CRP, C-reactive protein; ESR, erythrocyte sedimentation rate; ALT, alanine transferase; ULN, upper limit of normal. Bold values are statistically significant*.

### Treatment

Treatments intended for SARS-CoV-2 infection were prescribed in a half of patients, all hospitalized (368/759, 48.5%). The drugs most frequently used were macrolides (31.0%), followed by hydroxychloroquine (15.2%), lopinavir/ritonavir (5.7%) and remdesivir (1.4%). Systemic steroids were used in 28 cases (7.6%): 19 with COVID-19 complicated by MIS-C (67.9%), 7 with acute respiratory failure in pneumonia, 1 with myocarditis and 1 with severe pneumococcal sepsis.

Anticoagulant therapy was given in 24 cases (6.5%).

Low-flow oxygen-treatment was administered to 44 children (5.8%), of these 18 (41.0%) inside the ICU.

### Outcome

All but three children recovered. Two children died: one with cardiomyopathy and a metabolic disorder, the other with severe heart disease and esophageal atresia. One child recovered with sequelae (persistent coronary aneurysms following MIS-C).

No recurrence of infection was reported so far.

### Prolonged Viral Detection in Respiratory Samples

Among the 434 children for whom repeated SARS-CoV-2 PCR testing result was available, 65 (15.0%) had a prolonged positivity of respiratory samples ranging from 14 to 46 days from the first positive result. Viral load could not be estimated as the Ct value was not available for all children. No significant association was found between persistent positivity and gender, multiple contacts within the household, disease severity, immunocompromised state, underlying chronic diseases and laboratory parameters. Younger children showed a tendency toward a more prolonged detection of viral RNA in respiratory samples, although without statistically significant difference.

## Discussion

Our multicenter study is, to the best of our knowledge, one of the largest cohorts on the characteristics of laboratory-confirmed SARS-CoV-2 infection in European children and points out some relevant issues on its evolution in the pediatric population.

First, given the inclusion of both pauci/asymptomatic children managed in the community setting and hospitalized children, it provides a quite realistic insight on the natural history of SARS-CoV-2 infection in the pediatric setting and a rough estimate of children requiring hospital admission or encountering complications.

In our infected children, the course of the disease was extremely variable, ranging from mild and benign forms to critical patterns. Approximately a half of children was hospitalized. The others were either entirely managed by general pediatricians/family doctors or evaluated in the emergency department and then discharged at home. The two groups (inpatients and outpatients) were similar as far as gender distribution is concerned, but varied for age distribution, comorbidities and clinical pictures.

Children admitted for COVID-19 were younger than outpatients: prematurity and age < 1 year were independent risk factors for hospitalization, but not for COVID-19-related complications or intensive care admission. This finding suggests that a consistent number of hospital admissions were not due to disease severity but rather to a prudent approach by clinicians toward selected groups of patients (i.e., ex-preterms, neonates), beyond the propensity for their families to seek in hospital medical advice. Furthermore, it emphasizes the need for a robust territorial healthcare system in order to prevent emergency department congestion (9).

Compared to outpatients, children admitted for COVID-19 had a higher percentage of underlying chronic diseases, predominantly congenital malformations and complex congenital syndromes.

Hospital stay was slightly longer during the first phase of the pandemic, probably reflecting the major uncertainties about the new disease and the initial difficulties in arranging quarantines.

Among children with symptoms, initial clinical manifestations were frequently non-specific and indistinguishable from those of other common viral infections and showed typical age-related patterns (10–12). Fever was the predominant feature, followed by cough, rhinitis and gastrointestinal disturbances. This is in contrast with what observed in other reports, where fever was less common (36–56%) (5, 13). Among inpatients, fever, abdominal disturbances, dyspnea and febrile seizures were more frequent than in outpatients and generally represented the reason for hospital admission.

Complicated forms accounted for about 38% of hospitalized cases.

Older children, although less frequently admitted to hospital than infants, were at higher risk of complicated or severe diseases requiring prolonged hospital stay, with clinical pictures resembling those typically seen in adults.

Children with co-morbidities were more likely to develop complications and to need intensive care and ventilatory support. Therefore, underlying chronic diseases are confirmed risk factors for severe COVID-19 also in children (3, 14). Pulmonary complications such as pneumonia, respiratory distress syndrome and acute respiratory failure, were the most encountered ones, but occurred less frequently than in adults. In some patients with gastrointestinal involvement, the differential diagnosis included acute bacterial infections for the concomitant presence of mesenteritis, appendicitis or pancreatitis. Seizures and neurological complications also occurred, but the involvement of the central nervous system was limited to sporadic cases.

Complicated forms also included (7.7%) exaggerated inflammatory responses potentially leading to shock and multiple organ failure and requiring intensive care support, identified as MIS-C (6, 8). Interestingly, some children developed MIS-C during the replicating phase of infection, suggesting that SARS-CoV-2 may trigger a sort of “cytokine storm” also in earlier stages. The clinical manifestations and the evolution of the disease did not differ between those with earlier onset and those with later pictures. Consistently with previous studies, a large number of our MIS-C cases had gastrointestinal symptoms, respiratory and/or cardiac impairment and shock, sometimes with initial similarities to Kawasaki Disease (12, 13, 15–20). Age between 5 and 9 years, the presence of fever or abdominal pain as well as leukocytosis or lymphopenia were risk factors for MIS-C development. In rare cases, MIS-C resulted in coronary aneurysms, suggesting that SARS-CoV-2 virus can induce an immune-mediated cardiac injury.

Co-infections may have a role in disease progression. In our study they were searched for in about one third of children, mostly hospitalized. Among viruses, Rhino/Enterovirus were the most frequent ones, while *M. pneumoniae* was the second most frequent bacteria; influenza virus was rare as the first SARS-CoV-2 epidemic in Italy coincided with the end of influenza season. In a systematic review, *M. pneumoniae* was the most common co-pathogen detected (58%), followed by influenza virus (11%) and respiratory syncytial virus (11%) (21). Similarly to another European study, in our cohort viral co-infections were risk factors for severe disease requiring ICU admission and ventilatory support (3). This observation might have implications for the winter seasons, when the circulation of other respiratory pathogens, such as influenza virus, is likely to increase.

There are limited data on laboratory findings in children with COVID-19. In our study, barely 60% of tested inpatients showed increased CRP levels. Alterations in Troponin-T and NT-pro-BNP were specific markers of cardiac dysfunction in complicated cases, mainly MIS-C. Our data confirm that a lower lymphocytes count, as well as a high CRP levels in hospitalized patients, could be predictive of severe cases and need for ventilator support.

A chest X-ray was performed in about 40% of patients and CT scan in < 4%. The low propensity of Italian pediatricians to perform radiological examinations derives from the attempt to reduce X-ray exposure in childhood. Moreover, as radiological findings in children are not specific to COVID-19, X-ray or CT-scan are useful only to detect pulmonary complications. In this context, chest ultrasound may play an important role, although it was rarely performed in participating centers.

The great majority of hospitalized children with COVID-19 only required supportive therapy, including additional oxygen supplementation and fluid intake; only a bunch of patients were treated with antiviral or immunomodulatory drugs. Currently, most therapies remain experimental and their efficacy and safety are still debated. The role of these treatments should be better addressed through pediatric targeted clinical trials.

Our study confirms that SARS-CoV-2 infection is less aggressive in pediatric age than in adulthood and that case fatality rate in children and adolescents is very low (4, 22–24). In our series, all but three children completely recovered from the disease. Several hypotheses have been postulated to explain why children are less prone to severe COVID-19, including better immune response, lower prevalence of co-morbidities, higher mucosal colonization by other microorganisms interfering with the replication of SARS-CoV-2, lower expression of angiotensin converting enzyme-2 receptors and cross-protection by antibodies through common viral infections (25, 26). The proportion of asymptomatic children included was comparable to what emerged from recent reviews; however, it presumably underestimates the true percentage of asymptomatic cases, as these are less likely to be identified, unless screened within contact-tracing or specific programs (21, 27). The inclusion of asymptomatic outpatients in our population probably explains the more optimistic results compared with other studies considering only inpatients (10, 28).

We observed a higher, though not statistically significant, prevalence of infection in males in all age groups, similarly to other pediatric studies, supporting the hypothesis that sex-linked genetic factors may influence susceptibility to SARS-CoV-2 infection (5, 10, 29). However, gender did not affect disease severity.

Concerning the source of infection, children had frequently a contact history with infected households, especially mothers. This is consistent with previous reports, but could reflect the epidemiological status of the first phase of epidemic in Italy, when school and sport activity closure had minimized contact opportunities outside home (3, 24, 30). Interestingly, children with more than one infected household were less prone to develop complications and severe diseases. Further data are needed to better explain this finding, although a possible decreased pathogenicity of second/third line generation viruses might be supposed (4, 26).

The contribution of children to disease transmission is still under discussion. Recent findings suggest that those < 5 years with mild/moderate COVID-19 have higher amounts of SARS- CoV-2 RNA in their nasopharynx than older people (31). Although we did not perform quantitative assays, a relevant proportion of children had a prolonged detection of viral RNA in respiratory samples, regardless of symptoms or severity of disease. This may reflect a prolonged shedding of non-replicating viral particles, and may not affect contagiousness.

Our study has several limitations. First, the age limit at 18 years (childhood limit in Italy) makes results difficult to compare with papers with other age ranges. Secondly, enrolled children may not be fully representative of the Italian pediatric population affected, as our analysis includes a majority of hospitalized children and few cases managed at home. Finally, the limited number of selected subgroups of patients (i.e., those with cystic fibrosis or immunological defects) doesn't allow to draw conclusions in these settings.

In conclusion, complications of COVID-19 in children are related to the presence of co-morbidities. The length of hospital stay and the risk of complications increase with age: thus, the older the child, the closer the infection may resemble the adult one. Viral co-infections are additional risk factors for disease progression and high CRP levels and lymphopenia may be predictive markers of severe clinical pictures. Outside MIS-C, most children with COVID-19 require only supportive therapy.

## Data Availability Statement

The raw data supporting the conclusions of this article will be made available by the authors, without undue reservation.

## Ethics Statement

The studies involving human participants were reviewed and approved by Comitato Etico Interaziendale AOU Città della Salute e della Scienza di Torino – AO Ordine Mauriziano di Torino – ASL Città di Torino. Written informed consent to participate in this study was provided by the participants' legal guardian/next of kin.

## Author Contributions

SG, AL, LPiera, FC, FM, AM, EC, GV, DD, SB, ID, EV, EF, RG, MD, LPierr, GN, CMo, AK, CMa, P-AT, GP, ML, AV, and GC contributed to filling-in the registry forms on patient information. SG, GP, and MD were also responsible for data entry and elaboration. All authors, including those listed in the SITIP-SIP SARSCoV-2 pediatric infection study group, contributed to the conception of the work, the acquisition of data, critical revision of the intellectual content, and also read and approved the final version.

## Conflict of Interest

The authors declare that the research was conducted in the absence of any commercial or financial relationships that could be construed as a potential conflict of interest.
